# Multiplex Droplet Digital PCR Assay for Detection of *MET* and *HER2* Genes Amplification in Non-Small Cell Lung Cancer

**DOI:** 10.3390/cancers14061458

**Published:** 2022-03-11

**Authors:** Igor P. Oscorbin, Maria A. Smertina, Ksenia A. Pronyaeva, Mikhail E. Voskoboev, Ulyana A. Boyarskikh, Andrey A. Kechin, Irina A. Demidova, Maxim L. Filipenko

**Affiliations:** 1 Institute of Chemical Biology and Fundamental Medicine, Siberian Branch of the Russian Academy of Sciences (ICBFM SB RAS), 630090 Novosibirsk, Russia; mariasmr@mail.ru (M.A.S.); ks_pronyaeva@mail.ru (K.A.P.); taugepta@gmail.com (M.E.V.); boyarskih.u@gmail.com (U.A.B.); kechin@niboch.nsc.ru (A.A.K.); mlfilipenko@gmail.com (M.L.F.); 2 V. Zelman Institute for Medicine and Psychology, Novosibirsk State University, 630090 Novosibirsk, Russia; 3 Department of Natural Sciences, Novosibirsk State University, 630090 Novosibirsk, Russia; 4 Moscow City Oncology Hospital, 143423 Stepanovskoe, Russia; moldiag62@gmail.com

**Keywords:** non-small cell lung cancer, NSCLC, *MET*, *HER2*, gene amplification, digital PCR, multiplex ddPCR, real-time PCR, qPCR

## Abstract

**Simple Summary:**

Non-small-cell lung cancer (NSCLC) remains one of the most common tumors with a high mortality and morbidity rate. Alterations in *HER2* and *MET* could be a target for anti-tumor drugs or lead to resistance to anti-EGFR therapeutics. Existing methods for detecting *HER2* and *MET* amplifications are time and labor-consuming, and alternative methods are needed. We report the first multiplex droplet digital PCR (ddPCR) assay for the simultaneous detection of *MET* and *HER2* amplification in NSCLC samples. The suitability of qPCR was assessed for the optimization of multiplex ddPCR, and optimal ddPCR conditions were selected. The developed ddPCR was validated on artificial samples with various DNA concentrations and *MET* and *HER2* ratios. Using ddPCR, 436 EGFR-negative NSCLC samples were analyzed, and, among them, five specimens (1.15%) were *MET*-positive and six samples (1.38%) were *HER2*-positive. The multiplex ddPCR assay could be used for screening *MET* and *HER2* amplification in NSCLC samples.

**Abstract:**

Non-small-cell lung cancer (NSCLC), a subtype of lung cancer, remains one of the most common tumors with a high mortality and morbidity rate. Numerous targeted drugs were implemented or are now developed for the treatment of NSCLC. Two genes, *HER2* and *MET*, are among targets for these specific therapeutic agents. Alterations in *HER2* and *MET* could lead to primary or acquired resistance to commonly used anti-EGFR drugs. Using current methods for detecting *HER2* and *MET* amplifications is time and labor-consuming; alternative methods are required for *HER2* and *MET* testing. We developed the first multiplex droplet digital PCR assay for the simultaneous detection of *MET* and *HER2* amplification in NSCLC samples. The suitability of qPCR was assessed for the optimization of multiplex ddPCR. The optimal elongation temperature, reference genes for DNA quantification, and amplicon length were selected. The developed ddPCR was validated on control samples with various DNA concentrations and ratios of *MET* and *HER2* genes. Using ddPCR, 436 EGFR-negative NSCLC samples were analyzed. Among the tested samples, five specimens (1.15%) showed a higher ratio of *MET,* and six samples (1.38%) showed a higher ratio of *HER2*. The reported multiplex ddPCR assay could be used for the routine screening of *MET* and *HER2* amplification in NSCLC samples.

## 1. Introduction

Currently, lung cancer remains one of the most common malignancies. In 2020, lung cancer became the second most frequently diagnosed cancer, with 2.2 million estimated new cases (11.4% of all new cancer cases), and remained a leading reason for cancer-associated mortality, with an estimated 1.8 million deaths [1]. Thus, developing new lung cancer diagnostics and treatment approaches is a pressing challenge that can hardly be overestimated. Based on cell morphology and immunohistochemistry, lung cancer is divided into several subtypes: small cell lung cancer (15% of all cases) and non-small-cell lung cancer (NSCLC). NSCLC is further classified into the most common adenocarcinomas (63% of NSCLC), large-cell carcinomas (7% of NSCLC), and squamous cell carcinomas (18% of NSCLC) [2,3]. Over the past few decades, extensive studies revealed the genetic landscape of NSCLC, which allowed the identification of promising targets for therapeutic agents. Tyrosine kinase inhibitors against activated EGFR (epidermal growth factor receptor) [4] and ALK [5] are the most well-known such targeted drugs, widely used in routine clinical practice. Recent advances in the molecular oncology of NSCLC have broadened the spectrum of novel possible targets. Several of these genes—*ROS1*, *BRAF*, *NTRK1-NTRK3*, *MET*, *HER2*, *RET*, and *KRAS*—are high-potential candidates with several developing or approved inhibitors [6]. Thus, in 2021, FDA (U.S. Food and Drug Administration) approved sotorasib and amivantanab for the treatment of tumors harboring *KRAS* G12C and 20 exon insertions in *EGFR* genes, respectively [7,8]. It is believed that the list of available target drugs will be expanded more in the coming years against other mentioned gene products.

Among novel targets for inhibitors, *MET* (mesenchymal epithelial transition) and *HER2* (human epidermal growth factor receptor 2) have been extensively studied. *MET* and *HER2* alterations are also a reason for the resistance to commonly used anti-EGFR therapy. *MET* is an oncogene that encodes a receptor tyrosine kinase for hepatocyte growth factor (HGF, also known as scatter factor). The binding of HGF to the MET receptor activates downstream signaling pathways, such as RAS/ERK/MAPK and PI3K/AKT, and leads to increased cell motility and survival and enhanced angiogenesis [9,10]. MET overexpression is frequent in NSCLC (35–72%), whereas *MET* exon 14 skipping and *MET* amplification are relatively rare events (3–5% and 1–5%, respectively) [11]. The FDA has approved tyrosine kinase inhibitor crizotinib for NSCLC patients with MET exon 14 mutations [12]. For patients with MET-amplified NSCLC, crizotinib, capmatinib, and Sym015-01 are undergoing clinical trials [6]. Capmatinib and Sym015-01 showed potential as a cure for patients with NSCLC harboring *MET* amplification.

*HER2* is an oncogene encoding receptor tyrosine kinase ERBB2 that has no directly activating ligand. The constitutive activation of *HER2* promotes cell growth, proliferation, and survival [13]. HER2 is a well-known target for the treatment of breast cancer, with a long history of application of the respective anti-HER2 drugs [14,15,16,17]. HER2 overexpression is found in 2.8–38% of NSCLC cases, with a 1.5–4% frequency of mutations and 1.1–3% frequency of amplification [18,19]. Recently several anti-HER2 drugs have been assessed for patients with NSCLC [6]; the anti-HER2 antibody–drug conjugate trastuzumab–deruxtecan received FDA approval for NSCLC harboring *HER2* mutations [20]. Other anti-HER2 therapeutics are undergoing clinical trials.

Normally, for the detection of gene copy amplification events, academicians and clinical pathologists apply immunohistochemistry (IHC), fluorescence in situ hybridization (FISH), next generations sequencing (NGS), and multiplex ligation-dependent probe amplification (MLPA). However, all of these methods have limitations. IHC scoring could be subjective, FISH requires high-quality FFPE samples for testing, and NGS is amenable to contamination. Both techniques, as well as MLPA, are labor and time-consuming, demanding to the level of personnel training, relatively costly, and require a high input of the starting material for the analysis. The latter should be specifically noted; molecular pathologists often receive only biopsy samples from NSCLC patients, as no surgery is available. In addition, liquid biopsy, which is testing circulating tumor DNA in blood plasma, has recently been widely used in clinical practice. Liquid biopsy allows for the real-time monitoring of the appearance of molecular alterations leading to acquired resistance to target therapy. However, such testing requires sensitive and fast approaches.

Recently, various digital PCR techniques have been developed and used for numerous tasks, from somatic mutations and genetic modifications detection to the quantifying of viral genomic material. One of the possible applications of digital PCR is the testing of gene copy number events. Thus, numerous digital PCR-based approaches were reported for gene amplification detection for *MET* [21,22,23,24] and *HER2* [25,26,27,28,29,30] genes. However, most of these techniques are monoplex or duplex and require several separate reactions to analyze several genes. Different approaches for multiplexing digital PCR have been reported to address that problem based on the amplitude of end-point fluorescence or mixing of probes with different fluorophores. To date, no multiplex digital PCR has been published for *MET* and *HER2* gene amplification.

We report the first tetraplex ddPCR-based assay to detect *MET* and *HER2* gene amplification. The developed assay utilizes the amplitude of end-point fluorescence for multiplexing *MET* and *HER2* target genes and *RPP30* and *RAD51AP2* references genes. After optimization, we validated the ddPCR test on 436 NSCLC samples and proved its suitability for practical usage.

## 2. Materials and Methods

### 2.1. Clinical Samples and DNA Extraction

DNA was extracted from FFPE tumor tissue sections using QIAamp DNA FFPE Tissue Kit (Qiagen, Hilden, Germany) following the manufacturer’s protocol. FFPE sections were obtained from 436 NSCLC (lung adenocarcinoma) patients who have been operated on in 14 regional cancer centers across the Siberian and the Far Eastern Federal Districts of Russia.

### 2.2. Detection of Common EGFR Gene Mutations

Common *EGFR* mutations were analyzed using the cobas^®^ EGFR Mutation Test v2, Version 1 (Roche, Basel, Switzerland) according to the manufacturer’s instructions. The amplification procedure was conducted using cobas Z480 thermal cycler (Roche, Basel, Switzerland).

### 2.3. Positive Controls

Plasmids carrying *MET* and *HER2* genes fragments served as positive controls and were used to assess method sensitivity. All control plasmids used in this study were made in Shanghai RealGene Bio-tech, Inc. (Shanghai, China), purified, and sequenced (SB RAS Genomics Core Facility, ICBFM SB RAS, Novosibirsk, Russia). The purified plasmids were linearized using BamHI restriction endonuclease (SibEnzyme, Novosibirsk, Russia) and quantified using NanoDrop Lite A4 spectrophotometer (Thermo Fisher Scientific, Waltham, MA, USA) and droplet digital PCR.

### 2.4. Fluorescence In Situ Hybridization

Fluorescence in situ hybridization (FISH) was performed using a commercially available assay according to manufacturer recommendations. Briefly, 3 μm tissue section was hybridized overnight with the IVD MET (7q31)/SE7 (D7Z1) probe (Kreatech, Leica Biosystems, Lincolnshire, IL, USA). Normal tissue, including vessels, fibroblasts, lymphocytes, or non-neoplastic lung tissue, served as internal controls. Tumor tissue was entirely scanned for amplification hot spots by using a ×63 objective and appropriate filter sets (ZeissAxioimagerZ2 fluorescent microscope; Carl Zeiss, Oberkochen, Germany). Fifty cancer cells in four random areas were scored and interpreted using criteria reported by Camidge et al. [31].

### 2.5. Quantitative PCR

**Figure 1:**
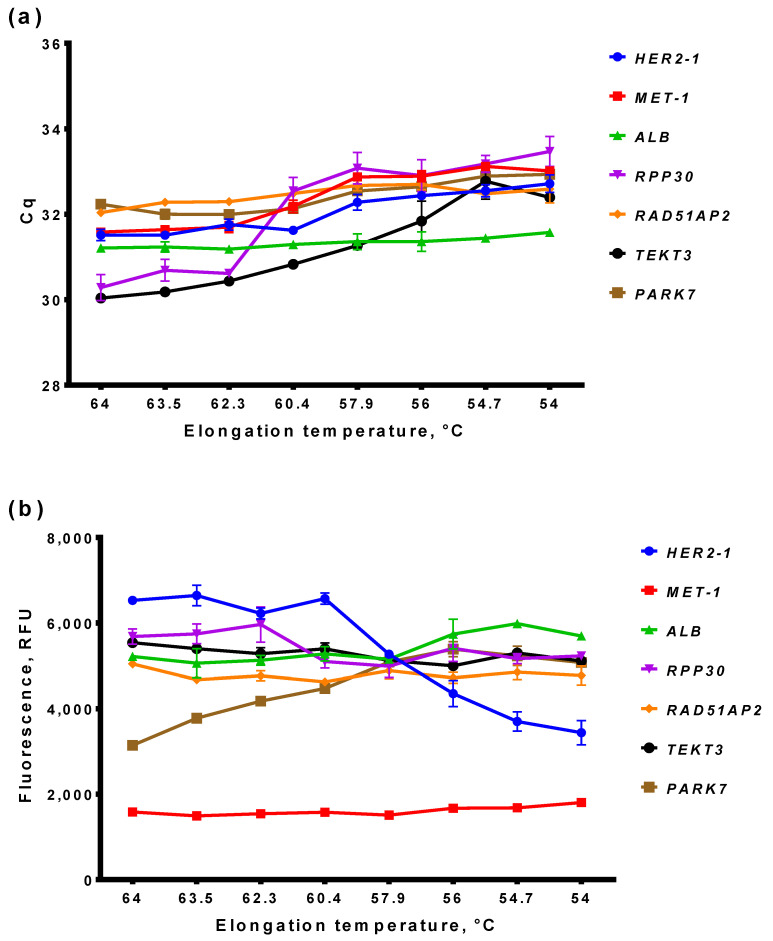
Results of qPCR with monoplexes. (**a**) Cq values; (**b**) end-point fluorescence after baseline subtraction. X-axis marks elongation temperature, Y-axis marks Cq values (**a**), or end-point fluorescence (**b**). Whiskers designate standard deviation in two experiments.

Quantitative PCR reactions were performed in 20 µL volume containing 65 mM Tris-HCl, pH 8.9, 24 mM (NH_4_) _2_SO_4_, 0.05% Tween-20, 3 mM MgSO_4_, 0.2 mM dNTPs, 900 nM primers, 250 nM TaqMan probe (Table 1), and 1 U of Taq-polymerase. Amplification was carried out in CFX96 Real-Time PCR Detection System (Bio-Rad, Hercules, CA, USA) according to the following program: 95 °C for 15 min followed by 45 cycles of 95 °C for 10 s, and 60 °C for 40 s with a collection of fluorescent signals at FAM and HEX channels. In the case of the elongation temperature gradient, this temperature was varied at the range of 54–64 °C. The acquired data were analyzed with CFX Manager software (Bio-Rad, Hercules, CA, USA) (Figure 1).

### 2.6. Droplet Digital PCR

The ddPCR assay was performed using the QX200 system (Bio-Rad, Hercules, CA, USA) according to the manufacturer’s recommendations. The reaction mixture in a volume of 20 µL consisted of 1× ddPCR master mix (Bio-Rad, Hercules, CA, USA), primers, probes, and DNA template. For quantification of standard samples, reactions contained 0.9 µM Lac-U/R primers, 0.25 µM Lac-P probe (Table 1), and approximately 10^4^–10^3^ copies of the tested plasmid standard. For the *MET* and *HER2* copy number analysis, concentrations of primers and probes were varied at the range of 450–900 and 125–250 nM, respectively; the exact concentrations are indicated in the respective sections. DNA amount of FFPE samples was in the range of 100–15000 copies per reaction (0.33–49.50 ng/reaction), and all specimens were analyzed in duplicates.

The entire reaction mixtures with 70 µL of droplet generation oil (Bio-Rad, Hercules, CA, USA) were loaded into a disposable plastic cartridge (Bio-Rad, Hercules, CA, USA) and placed in the droplet generator. After processing, the droplets obtained from each sample were transferred to a 96-well PCR plate (Eppendorf, Hamburg, Germany). The amplification was carried out using T100 Thermal Cycler (Bio-Rad, Hercules, CA, USA) according to the program: DNA polymerase activation at 95 °C for 10 min followed by 45 cycles of PCR amplification (94 °C for 30 s and 57 °C for 60 s), and 98 °C for 10 min, 2 °C/s ramp rate at all steps. After PCR, the droplets were counted with the QX200 Droplet Reader. The data obtained were analyzed with QuantaSoft Analysis Pro software (Bio-Rad, Hercules, CA, USA) and curated as explained in Section 3.2.3.

## 3. Results

### 3.1. Selection of References, Primers, and Probes

Here, we developed multiplex digital droplet PCR to test *MET* (7q31.2) and *HER2* (17q12) amplification events in FFPE samples from patients with NSCLC. For that purpose, we decided to quantify concentrations of target genes *MET* and *HER2* and two reference genes in a single tetraplex reaction. The multiplexing of ddPCR was based on the amplitude of end-point fluorescence. Thus, four independent amplification reactions were simultaneously performed in a single tube, two probes for two targets were FAM-labeled, and two probes were HEX-labeled. Probes with similar fluorophores were added to the reaction mixes at different concentrations, which resulted in a different end-point fluorescence for each target. Theoretically, up to 16 types of droplet clusters can be observed in such an assay, with various combinations of all four templates. The observed concentrations of targets allow for the calculation of a ratio between each target and the other three loci, and enable us to make a conclusion about the presence of *MET* and *HER2* amplification in the sample.

As candidates for references, several genes were selected based on published works. The criteria for candidate genes were the reported absence of registered copy number amplifications events in cancer cells or previous usage for human genomic DNA quantification. Previously, *EIF5B*, *DCK*, *RPS27A*, and *PMM1* genes were used as references to assess *HER2*, *MET*, and *EGFR* CNAs [24,30]. Here, we also tested reference genes on CNA in cancer cells using sequencing data from The Cancer Genome Atlas (TCGA) project. Several candidates met the mentioned requirements and were assessed for suitability in multiplex ddPCR: *ALB* (albumin, 4q13.3) [32,33], *RPP30* (ribonuclease P protein subunit P30, 10q23.31) [33,34], *RAD51AP2* (RAD51 associated protein 2, 2p24.2), *TEKT3* (tektin 3, 17p12), and *PARK7* (Parkinsonism associated deglycase, 1p36.23) [35].

Primers and probes for multiplex ddPCR were designed as described in our previous work [34]. Briefly, OligoAnalyzer software was used to select primers according to the following criteria: annealing temperature in the range of 60–62 °C for primers and 65–67 °C for probes, absence of G at the 5’-terminus of probes, the lengths of primers and probes in the range of 18–27 bases, Gibbs free energy ΔG of self-dimers ≥ −8 kcal/mol, and the amplicon length in the range of 61–100 bp. Sequences of selected oligonucleotides are listed in Table 1.

### 3.2. Optimization of Multiplex ddPCR Assay

#### 3.2.1. Optimal Elongation Temperature of ddPCR

After the selection of reference genes and oligonucleotides, designed sets of primers and probes were assessed in both quantitative PCR and droplet digital PCR simultaneously. The main purpose of optimization was proper droplet clustering in ddPCR, where all clusters can be clearly distinguished and no “rain” (droplets with intermediate fluorescence between two clusters) is observed. Currently, qPCR allows us to obtain results faster and with a lower cost than ddPCR. Therefore, we decided to test all primers and probes in qPCR and ddPCR in parallel to determine the suitability of qPCR for ddPCR optimization.

**Figure 2:**
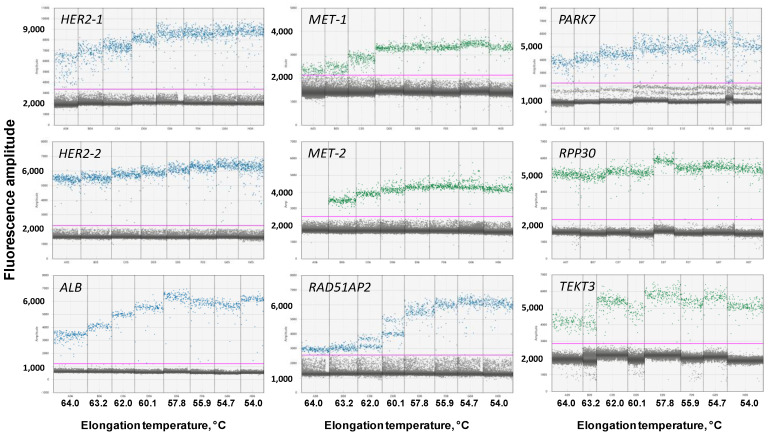
Results of ddPCR with monoplexes. Each panel represents results for the set of primers and probes that are designated at the left upper angle. X-axis marks elongation temperature, Y-axis marks end-point fluorescence.

At first, we determined the optimal elongation temperature for all primers and probes. For that, qPCR and ddPCR were performed with similar concentrations of primers and probes—900 and 250 nM, respectively—and DNA from white blood cells of a healthy donor was used as a template. The elongation temperature (T_elongation_) varied in the range of 54–64 °C. The results for qPCR are presented in Figure 1, and, for ddPCR, in Figure 2.

For all sets of primers and probes, the optimal T_elongation_ for qPCR was in the range of 64-60 °C, as minimal Cq-values and maximal end-point fluorescence were observed at these temperatures, indicating the highest qPCR efficacy. At a lower T_elongation_, the Cq-value increased for most primers and probes. However, in ddPCR, for most primers and probes, the highest end-point fluorescence was observed at temperatures lower than 58 °C, and a further decrease in T_elongation_ did not decrease the efficacy of ddPCR. No “rain” in ddPCR was observed at optimal temperatures for all primers and probes. Thus, the obtained results showed a discrepancy between the efficacy of qPCR and ddPCR at different T_elongation_. 

#### 3.2.2. Basic and End-Point Fluorescence in qPCR and ddPCR

**Figure 3:**
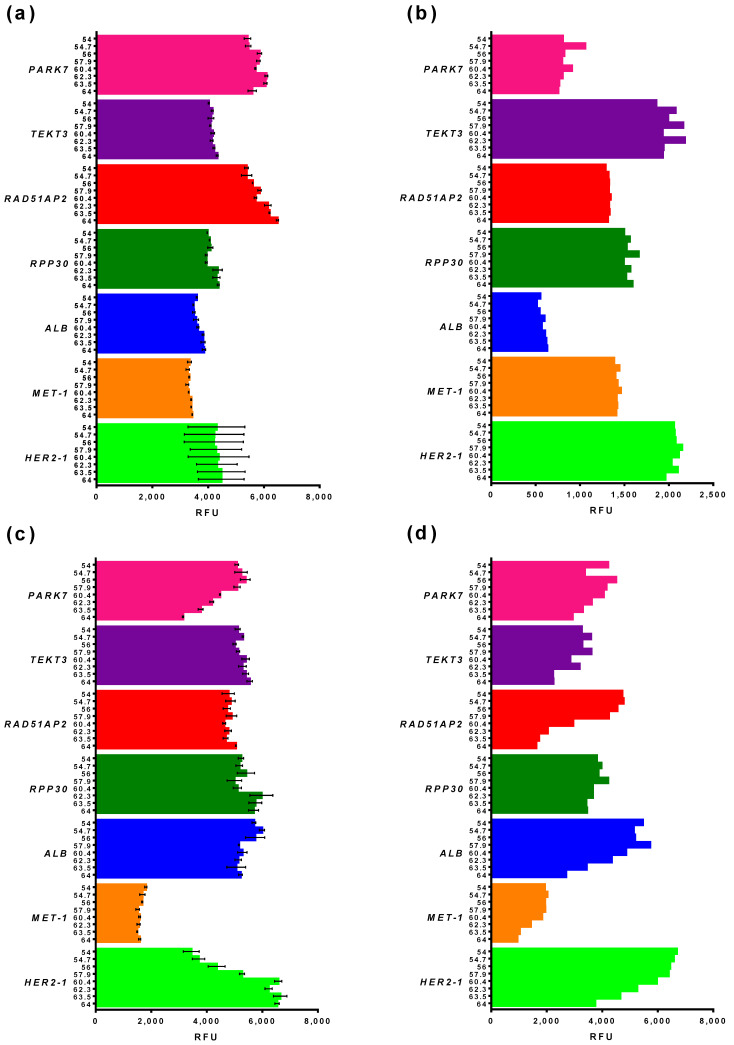
Fluorescence of monoplexes in qPCR and ddPCR. (**a**) Basic fluorescence of qPCR; (**b**) basic fluorescence of ddPCR; (**c**) end-point fluorescence after baseline subtraction of qPCR; (**d**) amplitude of end-point fluorescence ddPCR. Y-axis marks elongation temperature and monoplex, X-axis marks fluorescence in relative fluorescence units. Whiskers designate standard deviation in two experiments.

Simultaneously with the optimal elongation temperature, we compared the basic and end-point fluorescence of primers and probes in qPCR and ddPCR. These two parameters define the suitability of monoplexes for multiplexing, as high, basic, or low end-point fluorescence would hinder the combining of primers and probes in amplitude-based multiplex ddPCR. The results for qPCR are presented in Figure 3a,c, and, for ddPCR, in Figure 3b,d.

The basic fluorescence was different in qPCR and ddPCR (Figure 3a,b). Thus, the fluorescence of *PARK7* and *RAD51AP2* was the highest in qPCR, whereas, in ddPCR, the fluorescence of *TEKT3*, *HER2*, and *RPP30* was higher than *PARK7* and *RAD51AP2*. The fluorescence of *ALB* in qPCR was equal to *MET* and *RPP30*, whereas, in ddPCR, it was two times lower compared with the same loci. Therefore, qPCR data regarding the basic level of fluorescence could not be extrapolated directly in ddPCR.

Unlike basic fluorescence, the amplitude of end-point fluorescence is more stable between qPCR and ddPCR (Figure 3c,d). In most cases, this parameter was independent of T_elongation_ in qPCR, with the exception of *PARK7* and *HER2*, where end-point fluorescence was the highest at 64 and 54 °C, respectively. A direct correlation was observed between end-point fluorescence and T_elongation,_ as mentioned above. More importantly, the highest end-point fluorescence was almost the same for different loci, except for *MET*, whose end-point fluorescence was two to three times lower than for other loci. Notably, the ratio of end-point fluorescence of all loci was the same for qPCR and ddPCR, when *MET* was the lowest and *HER2* was the highest. Taken together, these results indicate that qPCR data cannot be directly used for the prediction of basic fluorescence levels in ddPCR, but are suitable for the forecasting of end-point fluorescence.

Preliminary tests showed that all primers and probes were suitable for ddPCR and can be used in further experiments.

#### 3.2.3. Reference Gene Selection

**Figure 4:**
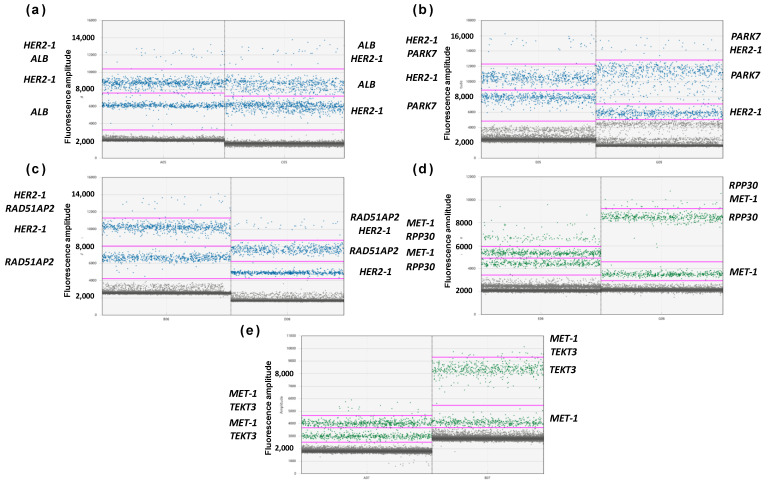
Results of ddPCR with duplexes. Each panel represents results for the set of primers and probes: (**a**) *HER2*-1, *ALB*, (**b**) *HER2*-1, *PARK7*, (**c**) *HER2*-1, *RAD51AP2*, (**d**) *MET*-1, *RPP30*, (**e**) *MET*-1, *TEKT3*. X-axis represents combinations of primers and probes with different concentrations, Y-axis represents end-point fluorescence. High concentrations of primers/probe correspond to 900/250 nM, low concentrations of primers/probe correspond to 450/125 nM. Clusters are designated at the sides of each panel.

After the assessment of elongation temperature and end-point fluorescence, primers and probes for different loci were duplexed to select the most favorable combinations of target and references genes. All primers and probes with similar fluorophores were mixed in two possible combinations, where the primers/probe for the target and reference were added to final concentrations of 900/250 nM and 450/125 nM. The results of combined duplexes are presented in Figure 4.

The purpose of duplexing was to achieve clearly distinguishable clusters for different sets of primers and probes. Therefore, for *HER2*, the best results demonstrated a combination of standard concentration *HER2* 900 nM primers and a 250 nM probe with *RAD51AP2* 450 nM primers and a 125 nM probe. With other combinations, clusters for different loci were closer to each other. Based on the same criteria, the best combination with *MET* was 450 nM primers and a 125 nM probe *MET,* and 900 nM primers and a 250 nM probe *RPP30*. However, the end-point fluorescence of *MET* was low, and the 900 nM primers and 250 nM probe were used in further experiments to facilitate the detection of *MET* clusters.

Notably, the results of duplexing were in agreement with basic and end-point fluorescence levels of monoplexes. Thus, the low end-point fluorescence of *MET* led to a poor clustering of duplexes with *MET*, where the fluorescence amplitude was insufficient for proper cluster identification. However, the observed rain in combinations of *HER2* and *PARK7* could not be predicted in respective monoplexes, which underlines the need to optimize multiplex ddPCR without skipping steps.

#### 3.2.4. Optimization of MET and HER2 Primers

**Figure 5:**
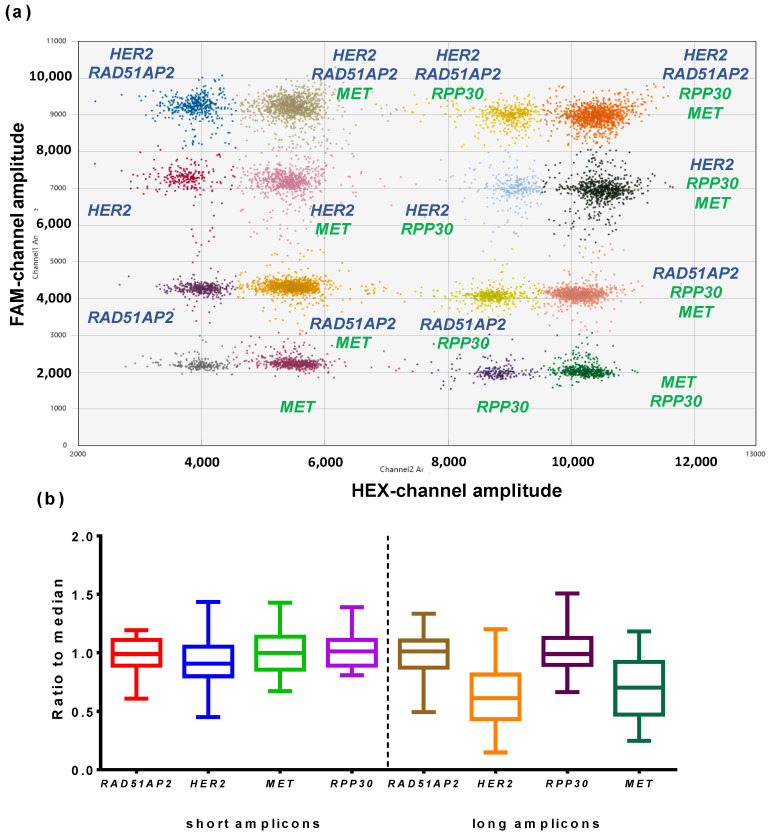
Results of pilot ddPCR with DNA from NSCLC FFPE samples. (**a**) 2D ddPCR plot. X-axis represents the amplitude of HEX-channel, Y-axis represents amplitude of FAM-channel. Loci are marked near respective clusters. (**b**) Tukey box-and-whiskers plot, X-axis represents genes, Y-axis represents the ratio of gene concentration to median concentration for both reference loci. Data for long amplicons are presented at the right side of the plot, data for short amplicons are presented at the left side.

At the last stage of optimization, we assessed the designed tetraplex ddPCR on a set of 46 DNA specimens purified from NSCLC FFPE tissue blocks. FFPE DNA samples are known to be highly fragmented and chemically modified, which leads to stalling DNA polymerase and a lower efficacy of PCR. Thus, the presence of rain, together with the actual DNA concentration for each target and reference loci, was appraised, and the ratio of concentrations between target and reference genes was calculated. The results of a pilot experiment with FFPE DNA samples are presented at Figure 5.

The designed tetraplex ddPCR showed clearly distinguishable clusters for all loci and was suitable for the analysis of DNA from FFPE samples; rain was moderate and did not hinder DNA quantification (Figure 5a). However, the observed ratio between the target genes (*HER2*, *MET*) and reference loci was close to 0.8, far from the expected ratio close to 1 (Figure 5b). The reason was that longer amplicons of *MET* and *HER2* were 100 bp, whereas amplicons of *RAD51AP2* and *RPP30* were 71 and 64 bp, respectively. To address this emerging issue, primers for target genes were redesigned with lower lengths: 72 bp for *HER2* and 77 bp for *MET*; the probes for target genes remained the same. New sets were validated in monoplexes and demonstrated the same efficacy of amplification (Figure 2). Thus, in further experiments, new primers for *HER2* and *MET* were used, and a close-to-1 ratio between the reference and target genes was observed (Figure 5b). Thus, the issue of underrepresented *HER2* and *MET* was successfully solved, and tetraplex ddPCR with shorter amplicons was used for further work.

### 3.3. Validation of Multiplex ddPCR Assay

#### 3.3.1. Fresh-Frozen Tissue and FFPE Sample in ddPCR

**Figure 6:**
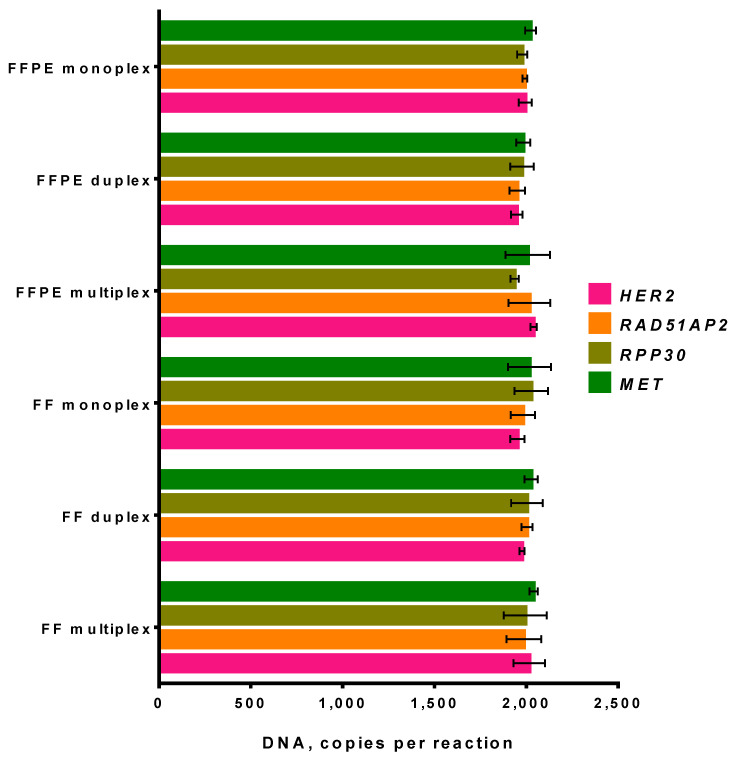
Influence of sample type on ddPCR performance. X-axis represents the concentration of genes; Y-axis shows the DNA sample and type of ddPCR—monoplex, duplex, or multiplex. FF—fresh-frozen, FFPE—formalin-fixed, paraffin-embedded samples. Whiskers designate standard deviation in two experiments.

Currently, the fixation of human tissues in a formalin solution followed by embedding them in paraffin is a common way to preserve a tumor sample for further studies. However, the process of sample fixation results in DNA fragmentation and the chemical modification of genomic DNA, such as the formation of crosslinks, cytosine deamination, and generation of abasic sites. These lesions can lead to DNA polymerase stalling anda reduction in DNA amplification efficacy dyeing PCR. To validate the suitability of the multiplex ddPCR assay for the FFPE samples analysis, we used matched fresh-frozen (FF) and FFPE samples from the single NSCLC tumor. DNA from matched FF and FFPE samples was purified and added in ddPCR with a concentration of 2000 copies per reaction, and all samples were tested in duplicate with the monoplex, duplex, and multiplex ddPCR assay (Figure 6).

The presented results indicate that, despite the degradation of DNA caused by the fixation process, DNA from FFPE samples remains suitable for the analysis. No difference in observed DNA concentrations was noticed for either monoplex, duplex, or multiplex analysis of fresh-frozen and FFPE sample. Thus, the developed multiplex ddPCR assay is suitable for the analysis of damaged DNA from FFPE samples.

#### 3.3.2. Influence of DNA Concentration on the Performance of ddPCR

The amount of DNA in a reaction is one of the crucial variables that define the sensitivity of testing. A low DNA concentration in a sample, i.e., close to dozens of molecules per microliter, leads to the stochastic presence of DNA molecules in the ddPCR reaction and possible false results of the analysis. A high concentration of DNA, i.e., more than 10^4^ molecules per microliter, results in an overload of ddPCR and a greater technical error with incorrect DNA quantification.

Another problem that could arise during the testing of FFPE samples is the ratio of amplified genes differing from whole numbers. Both the percentage of tumor cells and the exact degree of gene amplification can vary in a broad range in the analyzed specimen, leading to various observed gene amplification degrees. In that sense, the assessment of the minimal detectable gene amplification rate becomes necessary to determine the assay’s overall performance.

**Figure 7:**
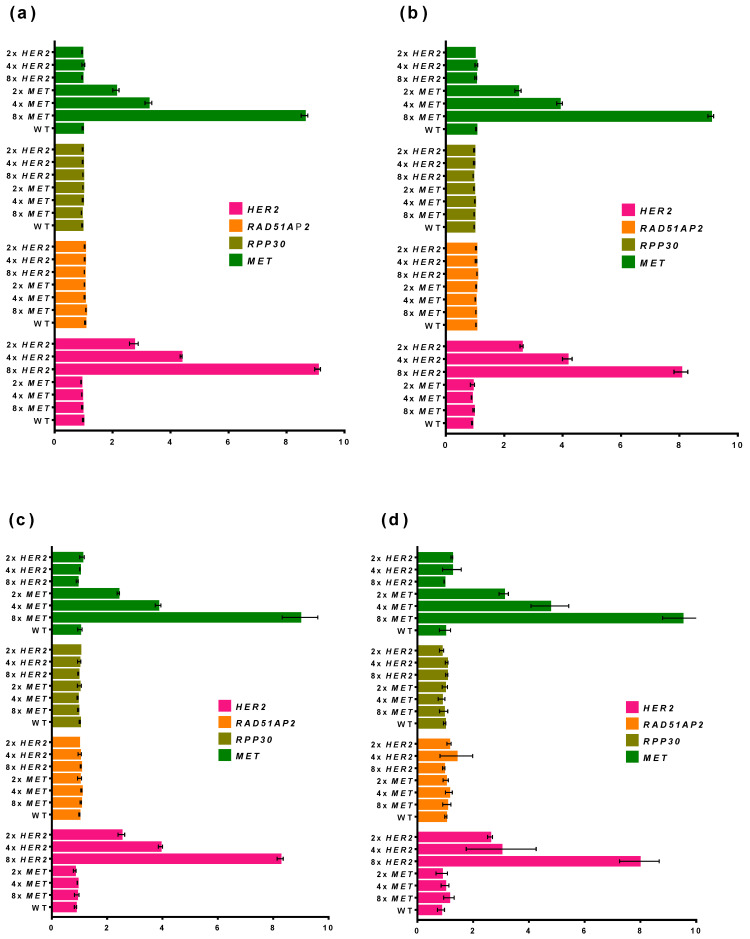

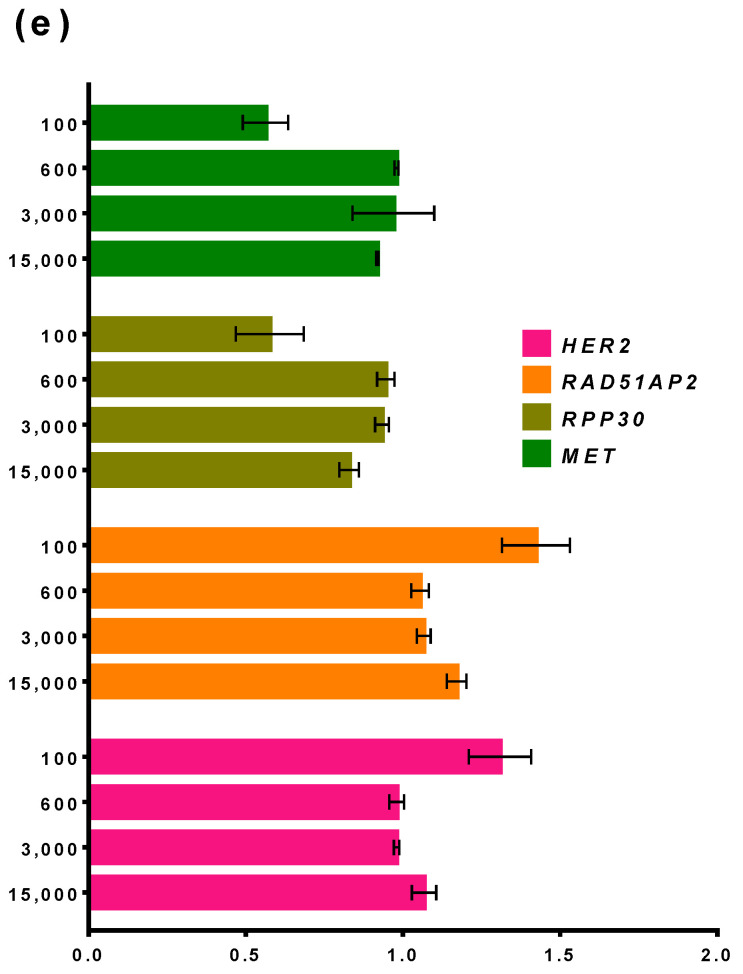
Influence of DNA amount on ddPCR performance. (**a**) 15,000 copies per reaction; (**b**) 3000 copies per reaction; (**c**) 600 copies per reaction; (**d**) 100 copies per reaction; (**e**) FFPE NSCLC sample titration. Each specimen was analyzed in duplicate. X-axis represents the ratio of target or reference gene concentration to normalized concentration of both reference genes, and Y-axis shows the DNA sample. Whiskers designate standard deviation in two experiments.

To assess the effect of the DNA concentration on the ddPCR performance, we prepared a set of samples with a varied ratio of *MET* and *HER2* genes (2×, 4×, 8×) and different DNA concentrations. The samples were prepared by mixing genomic DNA from white blood cells of a healthy donor with linearized plasmids carrying fragments of *MET* and *HER2* genes. The concentration of plasmids was measured with ddPCR and β-lactamase as a target for DNA quantification. Control samples were analyzed using the developed multiplex ddPCR-based assay. DNA was added in the reactions in the range of 100–15,000 copies per reaction. Simultaneously, the wild-type FFPE sample from the NSCLC tumor was titrated in ddPCR in the same range of DNA concentrations. All samples were tested in duplicate (Figure 7).

The copy number ratios were calculated by normalizing an amplicon copy number by the median value for all sample values and both reference values. To calculate the p-value, we evaluated the probability of meeting the normalized value for the same target among other samples with not less (for an amplification) or not more (for a deletion) than the value for a target considered using Equation (1):

(1) $$p-value=\frac{M_{x\geq x_{i}/x\leq x_{i}}}{N},$$

where $M_{x\geq x_{i}/x\leq x_{i}}$ is the number of samples for which the same target has the same or a higher (for amplification) or a lower (for deletion) copy number compared to both reference genes; *x* is the number of a sample; *N* is the total number of samples for which the current target has a high or low enough copy number.

For all samples, when 15,000, 3000, or 600 copies of DNA were loaded in reactions, the elevated ratio of *MET* or *HER2* genes was clearly distinguishable. No higher presence of target genes was detected when no respective plasmid was added to the sample. The observed ratios of *MET* and *HER2* genes were also close to the intended values, and calculating the ratios for the target genes was possible. The ratios of reference genes *RAD51AP2* and *RPP30* were also close to 1. When 100 copies of the template were analyzed, the SD of the ratio estimation increased and 2× and 4× *HER2* ratios became indiscernible. However, even in that case, *MET* or *HER2* amplifications were detectable. The titration of a wild-type FFPE NSCLC showed that the ratios of genes become unstable and varied at the range of 0.5–1.5, possibly hindering the CNA detection. Thus, the developed multiplex ddPCR assay can be used for the analysis in the range of DNA amount of 600–15,000 copies per reaction, and for the testing of FFPE samples with a low DNA concentration.

#### 3.3.3. Testing of MET-Positive Sample

**Figure 8:**
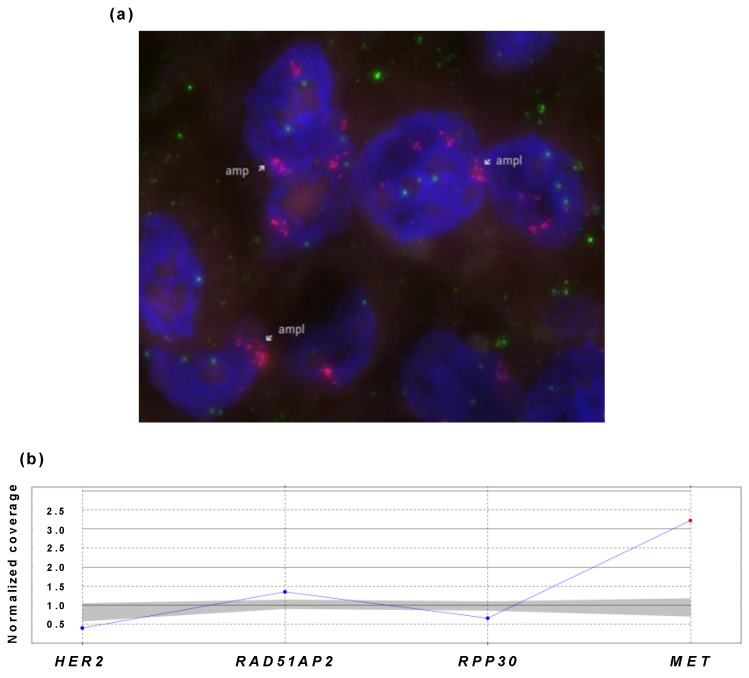
Results of *MET*-positive NSCLC sample analysis using FISH and ddPCR. (**a**) FISH assay. Green dots correspond to D7Z1 probes marking the chromosome 7 centromere, red dots correspond to probes marking the *MET* gene region at 7q31. (**b**) Multiplex ddPCR assay. X-axis shows genes, and Y-axis represents the ratio of gene concentration to normalized concentration of both reference genes.

To validate the multiplex ddPCR on a real FFPE DNA sample, we used a DNA sample from a patient with NSCLC and found *MET* amplification. *MET* amplification was detected using a commercially available IVD MET (7q31)/SE7 (D7Z1) probe in accordance with the manufacturer’s instructions (Figure 8a). Consequently, the sample was analyzed using the developed ddPCR assay (Figure 8b).

*MET* amplification levels were defined on the basis of the MET-to-CEP7 ratio—low, greater than or equal to 1.8 and less than or equal to 2.2; medium, greater than 2.2 and less than 4.0; and high, greater than or equal to 4.0. The case showed heterogeneous distributions of MET signals, with 50% of cells without signs of amplification and 50% with high amplification levels greater than 5.0.

The multiplex ddPCR assay revealed *MET* amplification in the tested sample with a ratio of the *MET* gene to the reference *RAD51AP2* and *RPP30* genes equal to 3 (*p* ≤ 0.025). Therefore, the results of the ddPCR assay were confirmed by FISH, giving a *MET* amplification degree in the range of 3–5. The developed ddPCR was proven to be suitable for *MET* amplification detection, and we proceeded to test NSCLC DNA samples.

### 3.4. Testing of Clinical Samples

**Figure 9:**
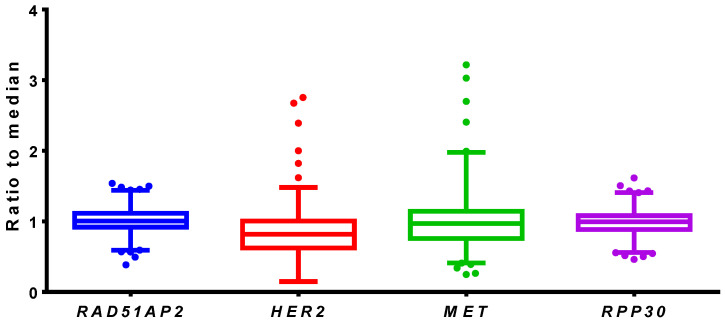
Results of NSCLC FFPE sample analyses by ddPCR. Tukey box-and-whiskers plot, X-axis represents genes, Y-axis represents the ratio of gene concentration to median concentration for both reference loci.

The validated multiplex ddPCR assay was used to analyze 436 *EGFR*-negative DNA samples from NSCLC FFPE tissue blocks. The samples were collected from patients from Siberia and the Far East of Russia and blinded before the analysis to hide the personal data of the patients. The EGFR status was determined using commercial qPCR-based kits. For each sample, the ratio of concentrations between target and reference genes was calculated. The obtained results are presented in Figure 9.

Among 436 tested NSCLC samples, five specimens (1.15%) showed a significantly higher ratio of *MET* to both reference loci (*p* ≤ 0.025), and six samples (1.38%) demonstrated a higher ratio of *HER2* (*p* ≤ 0.05). The increased ratio of *MET* was in the range of 1.92–3.22, and the increased ratio of *HER2* was in the range of 1.62–2.76. Keeping in mind the 15% common *EGFR* mutations rate in the studied population, the overall rate of *MET* and *HER2* amplifications was 0.98% and 1.12%, respectively.

It should be noted that, in five samples, the *MET* ratio was in the range of 0.25–0.41, which can be a result of bad clustering or a low DNA concentration in the respective samples. Another possible explanation is the presence of somatic mutations in primers and probe sites, leading to the observed efficacy of amplification.

Interestingly, a higher ratio of *RAD51AP2* (1.38–1.54) and a higher ratio of RPP30 (1.32–1.43) were observed in six and five samples, respectively. Such an increased ratio could be an analysis artifact caused by the selective inhibition of amplification due to the presence of contaminants in the tested samples or insufficient clustering. Another possible explanation is the presence of true amplification events involving reference genes in a small fraction of tumor cells. All samples with abnormal ratios of either target or reference genes were repeatedly analyzed using duplex ddPCR with one target and one reference gene. In all cases, the results of multiplex ddPCR were confirmed and similar ratios were obtained with duplex ddPCR (date not shown).

## 4. Discussion

The rapid growth of available and promising target drugs challenges modern molecular diagnostics in oncology. Emerging therapeutics demand the development of tests for the detection of respective target molecular alterations. Currently, some anti-MET and anti-HER2 drugs are undergoing clinical trials and require the design of appropriate molecular tests for *MET* and *HER2* amplification. *MET* [36,37,38,39] and *HER2* [40] alterations are also a reason for the resistance to commonly used anti-EGFR therapy. Existing approaches for *MET* and *HER2* testing, such as IHC, FISH, or NGS, could be subjective [41,42], time- and labor-consuming, costly, or, in the case of NGS, are not fully implemented for practice. An additional issue is the low amount of starting material: as in the case of NSLSC, FFPE samples are often prepared from tumor biopsies. Thus, alternative methods are needed to address the mentioned problems. Digital PCR is a sensitive method requiring a low amount of DNA that gives precise information about the DNA concentration. The multiplexing of digital PCR further reduces labor and reagents costs. Therefore, multiplex digital PCR could be used to facilitate the molecular diagnostics of NSCLC. Numerous reports described monoplex or duplex ddPCR for the detection of *MET* and *HER2* amplification in plasma and FFPE samples. However, to our knowledge, no multiplex ddPCR was designed for the simultaneous testing of *MET* and *HER2* copy number alterations (CNAs).

In the present work, we applied the amplitude-based approach for the multiplexing of digital PCR. This approach relies on using probes with the same fluorophore for the detection of different targets. The probes are added to the reaction in different concentrations, resulting in a difference in final fluorescence and clustering. This strategy is widely used to design multiplex ddPCR, and was successfully applied by us to detect *BRCA1* gene copy number variations.

To optimize reaction conditions for multiplex ddPCR, we tested the optimal elongation temperature and fluorescence levels of primers and probes for the target *MET* and *HER2* gene and a number of possible reference genes. Simultaneously with digital PCR, primers and probes were tested in quantitative PCR; the latter method is less time-consuming and costly. Thus, we assessed the possibility of extrapolating the results of qPCR on ddPCR for speeding up and lowering the cost of ddPCR optimization.

A direct comparison of qPCR and ddPCR results gave contradicting results. Thus, the decrease in qPCR efficacy at a lower elongation temperature did not correlate with the efficacy of ddPCR. Another important variable, the basic fluorescence level in ddPCR, cannot be predicted using qPCR. The ratio of basic fluorescence between different primers was different in qPCR and ddPCR, resulting in an impossible direct prediction from qPCR data. It should be recalled that primers and probes with a high, basic, and low end-point fluorescence can be obtained using qPCR and do not have to be used for ddPCR, saving time and effort. End-point fluorescence was more stable between qPCR and ddPCR; the ratio of fluorescence between different targets was similar in qPCR and ddPCR. Thus, end-point fluorescence in ddPCR can be forecasted based on qPCR. To sum up, qPCR has a limited predicting ability for the determination of ddPCR optimal conditions. It allows us to predict end-point fluorescence for various targets, but has reservations in the farseeing of the optimal elongation temperature and basic fluorescence. Therefore, the testing of primers and probes in ddPCR cannot be skipped and replaced by qPCR.

Several targets were chosen as two possible optimal references. Such precautions were made to prevent potential issues with a lower PCR efficacy after multiplexing, as primers for different targets can interfere and affect clustering in ddPCR. After testing in monoplexes, primers for different loci were tested in duplex ddPCR. Theoretically, data on the optimal elongation temperature and fluorescence from monoplexes are enough to calculate optimal primer and probe concentrations for multiplexes, and a duplex test is not necessary. However, primers from different sets can be incompatible in a single reaction, as was mentioned above, and this possibility can only be assessed in an actual experiment. Here, results for almost all combinations of reference and target genes were in accordance with the data from monoplexes. However, *HER2*/*PARK7* combinations demonstrated rain in a duplex that was not observed in the respective monoplexes. This notion underlines the need for the prior duplex testing of different primer combinations before combining them in a more complex multiplex.

At the final step of optimization, the resulting tetraplex assay was validated on a small set of real FFPE samples to determine the actual performance ddPCR. It was observed that the target loci ratio was lower than that for the reference gene. The possible reason behind the underestimation was the length of target gene amplicons, which was 30 bp longer than the reference loci. It is well-known that DNA from FFPE samples is highly fragmented and chemically modified. Here, it led to the underestimation of target genes due to a lower concentration of longer DNA template molecules. After introducing shorter amplicons, the ratio for *MET* and *HER2* genes became close to theoretical 1, supporting the hypothesis about the influence of DNA amplicons. This observation highlights the need for a careful primer design for the amplification of FFPE samples when the length of amplicons can significantly affect the actual performance. Commercial assays for different targets should be assessed with special precautions, as the length of amplicons could be unknown. Not matching amplicon lengths can result in biased data, especially for CNA detection. 

The developed multiplex ddPCR assay was successfully validated using a MET-positive NSCLC sample and a set of 436 EGFR-negative samples. Thus, ddPCR confirmed the presence of *MET* amplification in the sample tested by FISH. After correction on the frequency of common EGFR mutations, the observed rates of *MET* and *HER2* amplifications were 0.98% and 1.12%, respectively. These findings are in good agreement with previous studies, demonstrating a similar 1–5% frequency of *MET* and *HER2* CNAs. Several works also reported the coexistence of *EGFR* mutations with various *MET* and *HER2* alterations; however, those events were rare [19]. For that reason, no EGFR-positive samples were tested here.

Several limitations of the present study should be mentioned. The set of FFPE samples used for the validation of the developed ddPCR assay was relatively small. The insufficient number of tested NSCLC specimens could lead to an erroneous estimation of actual *MET* and *HER2* amplification rates. Only a single sample was validated as MET-positive using gold standard FISH, while a poor correlation between FISH and ddPCR was once reported [43]. 

Droplet digital PCR also has some reservations. If handled without proper care, ddPCR could be contaminated by amplicons produced in previous runs. Contamination leads to biased results and requires substantial efforts for elimination. Therefore, the ddPCR workflow should be organized in the one-way movement of samples and should prevent any possible contacts of ddPCR reagents with samples and consumables after amplification. Digital PCR is a targeted method that could detect only known mutations, whereas NGS allows for the detection of all alterations in analyzed genomic sites. Expanding the spectrum of novel targets makes NGS promising for the simultaneous detection of all possible mutations with available target drugs. Targeted methods require numerous separate reactions to test many mutations, whereas NGS allows us to analyze all of them in a single run. However, ddPCR remains more sensitive than NGS and can clarify the results of a preliminary screening made by NGS or other methods. A high sensitivity of the analysis can be crucial in the case of liquid biopsy testing, where extremely low amounts of tumor DNA could be present in the studied samples. Therefore, ddPCR could be applied for circulating tumor DNA analysis, taking a sensitive approach for analyzing samples with a low amount of tumor DNA. Monoplex ddPCR assays were successfully validated previously, making it possible to develop more complex multiplex ddPCR techniques.

To sum up, the developed ddPCR-based assay was optimized using both qPCR and ddPCR data and validated on samples with various DNA concentrations and with the *MET* and *HER2* amplification degree. The assay was used to test 436 *EGFR*-negative NSCLC samples. The observed rates of *MET* and *HER2* amplification were in agreement with previous studies.

## 5. Conclusions

Here, we report the first multiplex droplet digital PCR assay for the simultaneous detection of *MET* and *HER2* amplification in NSCLC samples. The suitability of qPCR was assessed for the optimization of multiplex ddPCR. The optimal elongation temperature, reference genes for DNA quantification, and amplicon length were selected. The developed multiplex ddPCR was validated on control samples with various DNA concentrations and ratios of *MET* and *HER2* genes, and on a *MET*-positive NSCLC sample. Using a ddPCR-based assay, 436 *EGFR*-negative NSCLC samples were analyzed. Among the tested samples, five specimens (1.15%) showed a significantly higher ratio of *MET* (*p* ≤ 0.025), and six samples (1.38%) demonstrated a higher ratio of *HER2* (*p* ≤ 0.05). The increased ratio of *MET* was in the range of 1.92–3.22 and the increased ratio of *HER2* was in the range of 1.62–2.76. The multiplex ddPCR assay reported here could be used for a routine screening of *MET* and *HER2* amplification in NSCLC samples.

## Figures and Tables

**Table 1:** Sequences of primers and probes.

Gene, Amplicon Length	Oligonucleotide	5′-Sequence-3′
*HER2* set 1 100 bp	HER2-F1	CATCTTCCACAAGAACAACCAG
	HER2-R1	GTCTGAGAGAAGAGGCAGCA
	HER2-P	FAM-AGACACCAACCGCTCTCGGGC-BHQ1
*HER2* set 2 72 bp	HER2-F2	CAGCTGGCTCTCACACTGA
	HER2-R2	AAGAGGCAGCAGGGAGG
	HER2-P	FAM-AGACACCAACCGCTCTCGGGC-BHQ1
*MET* set 1 100 bp	MET-F1	AAGTGGGGAACTGATGTGACTTA
	MET-R1	CAAGATCGTCAACAAAAACAATG
	MET-P	HEX-CGGACCCAATCATGAGCACTG-BHQ1
*MET* set 2 77 bp	MET-F2	CAATGTGAGATGTCTCCAGCAT
	MET-R2	GGGAACTGATGTGACTTACCCTA
	MET-P	HEX-CGGACCCAATCATGAGCACTG-BHQ1
*ALB* 94 bp	ALB-U	GACTTGCCAAGACATATGAAACC
	ALB-FAM	FAM–TGCTGTGCCGCTGCAGATCC-BHQ1
	ALB-R	TCCAACAATAAACCTACCACTTTG
*PARK7* 74 bp	PARK7-F	CGTTGCAGGCCTGGCTGGAAA
	PARK7-R	CAAGGCTGGCATCAGGACAAATG
	PARK7-P	FAM-CCCAGTACAGTGTAGCCGTGATGTGG-BHQ1
*RAD51AP2* 74 bp	RAD51AP2-U	TGGTGACTTTTGCCCATATTAG
	RAD51AP2-R	GTGGTCAACAAAATACGTGCA
	RAD51AP2-P	FAM-CCTGCTATAGTATCATGGAACGAGG-BHQ1
*RPP30* 61 bp	RPP30-F	CAGATTTGGACCTGCGAGCG
	RPP30-R	GAGCGGCTGTCTCCACAAGT
	RP30-HEX	HEX-TCTGACCTGAAGGCTCTGCGCG-BHQ2
*TEKT3* 67 bp	TEKT3-F	GCGTGTGCCTGACTTACGTTG
	TEKT3-R	ACATCAGACGGTGTCGGCTAC
	TEKT3-P	HEX-CGACCCTCTCCACTCCGCG-BHQ2
Beta-lactamase	Lac-U	CGTCGTTTGGTATGGCTTCATTC
	Lac-R	AGGACCGAAGGAGCTAACCG
	Lac-P	HEX-CGGTTCCCAACGATCAAGGCGAG-BHQ2

## Data Availability

The data presented in this study are available on request from the corresponding author. The data are not publicly available due to ethical reasons.
